# *Staphylococcus aureus* Manipulates Innate Immunity through Own and Host-Expressed Proteases

**DOI:** 10.3389/fcimb.2017.00166

**Published:** 2017-05-05

**Authors:** Giampiero Pietrocola, Giulia Nobile, Simonetta Rindi, Pietro Speziale

**Affiliations:** Unit of Biochemistry, Department of Molecular Medicine, University of PaviaPavia, Italy

**Keywords:** *Staphylococcus aureus*, secreted virulence factors, innate immunity, immune evasion molecules, protease, host protease modulator

## Abstract

Neutrophils, complement system and skin collectively represent the main elements of the innate immune system, the first line of defense of the host against many common microorganisms. Bacterial pathogens have evolved strategies to counteract all these defense activities. Specifically, *Staphylococcus aureus*, a major human pathogen, secretes a variety of immune evasion molecules including proteases, which cleave components of the innate immune system or disrupt the integrity of extracellular matrix and intercellular connections of tissues. Additionally, *S. aureus* secretes proteins that can activate host zymogens which, in turn, target specific defense components. Secreted proteins can also inhibit the anti-bacterial function of neutrophils or complement system proteases, potentiating *S. aureus* chances of survival. Here, we review the current understanding of these proteases and modulators of host proteases in the functioning of innate immunity and describe the importance of these mechanisms in the pathology of staphylococcal diseases.

## Introduction

*Staphylococcus aureus* is a human pathogen known for its ability to cause both community- and nosocomial-acquired diseases ranging from mild skin infections, such as impetigo to severe diseases, such as endocarditis, pneumonia, sepsis and toxic shock syndrome (David and Daum, 2010). Treatment of *S. aureus* infections with antibiotics is often ineffective due to the development of antibiotic-resistance strains, such as methicillin-resistant S. *a**ureus* (MRSA). Therefore, alternative treatment options and vaccination are now being explored (Bagnoli et al., 2012; Pozzi et al., 2015). The success of *S. aureus* as a pathogen depends on the production of several virulence factors. *S. aureus* can express up to 24 cell wall-anchored proteins, which promote adhesion to extracellular matrices, invasion of non-phagocytic cells, biofilm formation (Foster et al., 2014) and interference with neutralization of the innate immune system (Sjodahl, 1977; Cary et al., 1999; Kang et al., 2013).

*S. aureus* also produces a wide variety of peptides that inhibit specific steps of the innate immune system, which represents the first line of defense of the host (Rooijakkers et al., 2005a; Itoh et al., 2010; Thammavongsa et al., 2015) (For more details see below).

Potentiation of *S. aureus* pathogenesis is determined by secretion of proteases that cleave specific components of the host immune system or disrupt the integrity of extracellular matrix and intercellular connections, compromising the stability of the host tissues and contributing to the dissemination of the infection (Koziel and Potempa, 2013). *S. aureus* also secretes proteins that can bind and modulate host protease precursors which, in turn, can target specific defense components, providing the bacterium with additional tools to establish colonization of the tissues (McAdow et al., 2012). Lastly, some *S. aureus* secreted molecules can bind and inhibit neutrophil serine proteases which are important for several functions including the regulation of extracellular trap formation (Hu, 2012; Kolaczkowska et al., 2015). Altogether, these findings highlight the relevance of these compounds as important virulence agents of *S. aureus* infections.

In this review, we focus on recent advances in the characterization of *S. aureus* proteases and modulators of host proteases, and their ability to avoid innate immunity. We also discuss how understanding the mechanisms of these immune evasive factors can have an impact in the development of therapeutics against *S. aureus* diseases.

## The innate immune system

The innate immune system is the collection of tissues, cells and molecules that protect the body from a variety of pathogenic microbes and toxins present in our environment. The innate immune system has numerous functions, including:

1. Action as anatomical barrier to infectious agents,
2. Activation of the complement cascade to identify bacteria, activate cells, and promote clearance of antibody complexes or dead cells,
3. Recruitment of innate immune cells that attack foreign cells to sites of body infection, through the production of chemical specialized factors or mediators called cytokines.

## The epithelial surface as the first line of defense against *S. aureus* infection

**Figure 1:**
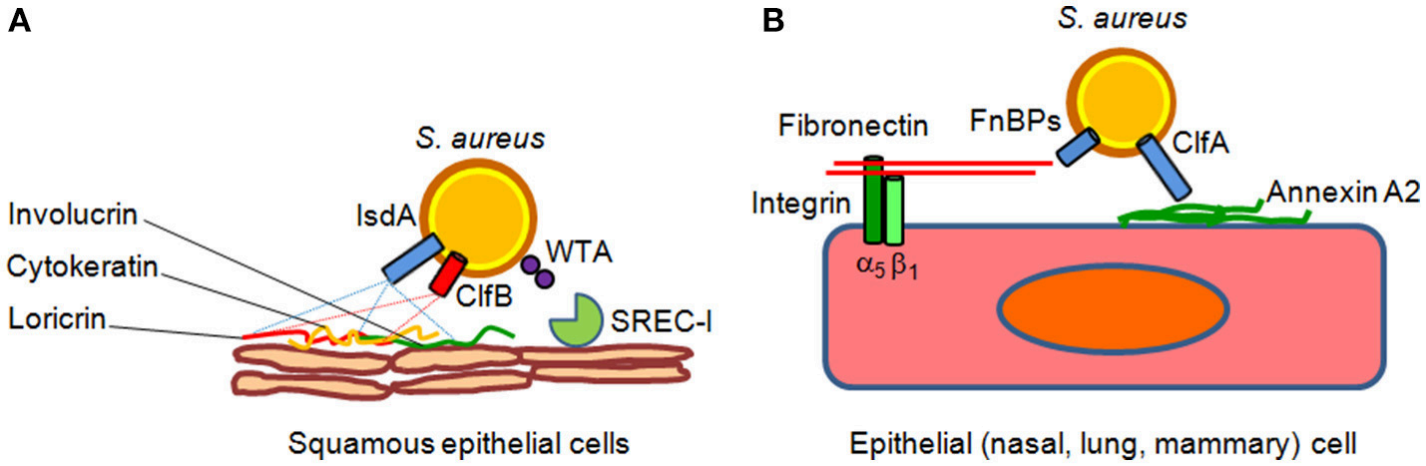
**Models of ***S. aureus*** adherence to and invasion of epithelial cells. (A)** Adherence of *S. aureus* to epithelial cell surface is mediated by clumping factor B (ClfB) through high affinity interactions with cytokeratin 10 and loricrin. Iron-regulated surface determinant A (IsdA) protein further contributes to epithelial adherence by binding to the cornified cell envelope protein loricrin, involucrin and cytokeratin 10. Wall theicoic acid (WTA) glycopolymer has been also proposed to promote staphylococcal adhesion through an epithelial type F scavenger receptor named SREC-I. **(B)** Fibronectin-binding proteins A and B bind to the extracellular matrix fibronectin which interacts with integrin α_5_β_1_ on the surface of epithelial cells, thereby triggering invasion of the cells. Recently, it has been suggested that clumping factor A (ClfA) bind to surface-associated annexin A2, and this interaction could mediate *S. aureus* internalization into mammary epithelial cells.

Intact epithelial surfaces form physical barriers between microbes in the external environment and host tissue. The main interfaces between the environment and the host are the skin and the mucosal surfaces of the gastrointestinal and respiratory tracts. Tight junctions between neighboring cells prevent easy entry by potential pathogens, such as *S. aureus*. The interior epithelial surfaces are also covered with a mucus layer that protects these surfaces against microbial, mechanical, and chemical insults. The slimy mucus coating, made primarily of secreted mucin and other glycoproteins, physically helps prevent pathogens from adhering to the epithelium. Epithelia also produce peptides that kill or inhibit the growth of pathogens. Those that are most abundant include antimicrobial peptides called defensins. They are generally short and positively charged, and have hydrophobic or amphipathic domains in their folded structure. Defensins are also the most abundant protein type in neutrophils, which use them to kill phagocytosed pathogens (Zhao and Lu, 2014). Besides the defensins, cathelicidins represent another family of antibacterial peptides in mammals (Boman, 2003; Brogden et al., 2003). The human cathelicidin hCAP-18, constitutively expressed by neutrophils and squamous epithelia in response to inflammatory challenge is processed by proteinase 3 to generate the active peptide LL-37 (Sørensen et al., 1997) that possesses considerable anti-staphylococcal activity (Tkalcevic et al., 2000; Travis et al., 2000). *S. aureus* uses several mechanisms to counteract the epithelia defense actions. Adhesion to epithelia is a multifactorial process that involves the host as well as bacterial factors. One key factor is the glycopolymer cell wall teichoic acid of *S. aureus*, which directly interacts with nasal epithelial surface through a type F scavenger receptor named SREC-I (Baur et al., 2014). Another important surface factor with a role in nasal and possibly skin epithelia colonization is the cell wall-anchored protein clumping factor B, which binds to fibrinogen, cytokeratin, the dominant component of the interior of squamous cells and loricrin, the most abundant protein of the cornified envelop of squames (Lacey et al., 2016). Iron-regulated surface determinant A protein also promotes the adhesion of *S. aureus* to squames cooperating in binding to cornified cell envelop loricrin, involucrin, and cytokeratin. Other cell wall-anchored proteins such serine-aspartate dipeptide repeat proteins SdrC, SdrD, and SasG promote adhesion to squames but their ligands are unknown (Foster et al., 2014; Figure 1).

Although *S. aureus* is not considered an intracellular pathogen, it can govern its uptake into non-phagocytic cells. Bacterial internalization is promoted by fibronectin-binding proteins A and B. Binding of fibronectin to fibronectin-binding proteins and its subsequent recognition by integrin a_5_b_1_ leads to internalization of the bacterium into epithelial and endothelial cells (Foster et al., 2014). Recently, it has been shown that clumping factor A binds annexin A2, a calcium-regulated membrane-binding protein, and it has been proposed that this interaction could also mediate *S. aureus* invasion into bovine mammary epithelial cells (Bonora et al., 2015; Figure 1). In lung epithelial cells *S. aureus* internalization also involves the efflux pump Tet38 via interaction with CD36 (Truong-Bolduc et al., 2015, 2017).

To disturb the defensive barrier function of the airway epithelium, *S. aureus* α-hemolysin disrupts cell-matrix adhesion by activating Fak signaling with the consequent acceleration of focal contact turnover (Hermann et al., 2015). Additionally, treatment of airway epithelial cells with recombinant α-hemolysin results in plasma membrane depolarization, and increased phosphorylation of paxillin and p38 MAP kinase, a signal transduction module involved in host defensive actions (Eiffler et al., 2016). Lastly, staphylococcal EsxA protein interferes with epithelial cell apoptotic pathways and, together with EsxB, mediates the release of intracellular staphylococci from the host cells (Truong-Bolduc et al., 2015).

## Complement system and its impact with *S. aureus* virulence factors

**Figure 2:**
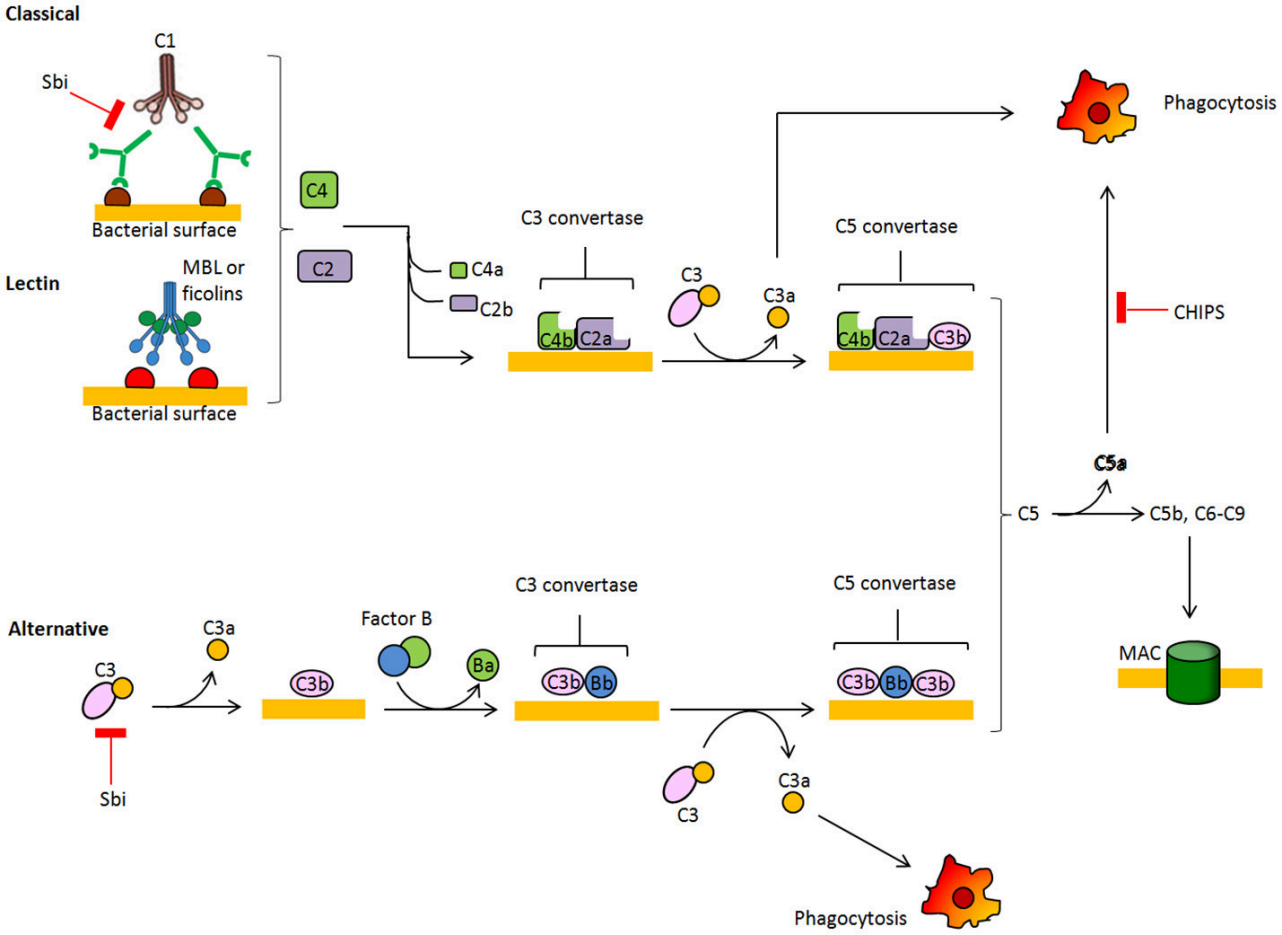
**Schematic overview of the complement system**. The complement cascade is activated by recognition of microbe-bound antibodies or bacterial sugars by C1 complex (CP, classic pathway) or the MBL and ficolin MASP-2 complex (LP, lectin pathway), respectively. Both C1 and MASP-2 cleave C4 and C2 to generate a C4b2a complex on the bacterial surface. This complex is a C3 convertase that cleaves C3 into C3a and C3b, which binds covalently to the bacterial surface. The alternative pathway (AP) C3 convertase C3bBb is generated after binding factor B to surface bound C3b and subsequent cleavage by factor D. C3b molecules also generate C5 convertases C4bC2aC3b and C3bBbC3b by binding near to C3 convertases. C5 convertases cleave C5 into soluble C5a, which attracts neutrophils to the site of infection, and C5b which forms a complex with C6-9 proteins to generate the membrane attachment complex.

During colonization and in the infection stage, *S. aureus* is faced with the host's innate immune defense, and one of the first barriers it encounters is the complement system. Several complement effector molecules can indeed sense and opsonize *S. aureus* cells and promote their phagocytic killing by neutrophils in blood and macrophages in tissues. The complement system is a proteolytic cascade of plasma proteins, which is crucial to the host's defense against invading bacteria (Figure 2). Complement fixation by bacteria can occur through three activation routes, the classic pathway (CP), the lectin pathway (LP), and the alternative pathway (AP). Activation of the CP starts after C1q molecules are deposited on the bacterial surface via direct binding, immunoglobulin recruitment, or pentraxins bridging and interacting with C1r and C1s proteases to form the C1 proteolytic complex. Through the LP pathway, collectins, such as mannose-binding lectin (MLB), mannan-binding lectin or ficolin, bind to microbial surface polysaccharides, resulting in activation of mannan-binding lectin-associated serine protease (MASP). Both CP and LP proteolytic complexes can split surface-bound C4 into C4a plus C4b, and C2 into C2b plus C2a protease. C4b and C2a directly combine and form the C3 convertase C4bC2a, which cleaves native C3 into C3b and C3a. C3b molecules effectively opsonize the bacterium and facilitate activation of C3bBb convertase, the AP convertase that promotes transformation of new C3 molecules into C3b and C3a, thus amplifying the number of C3b molecules that opsonize bacteria and promote phagocytic killing. Surface-tethered C3b also plays a central role in the formation of the two C5 convertases (C4bC2aC3b and C3bBbC3b), which cleave C5 into C5a and C5b. C5b initiates the assembly of the membrane attack complex (MAC), a pore made up of components C5b, C6, C7, C8, and multiple units of C9 and ultimately leading to cell lysis. Important host regulators controlling complement homeostasis are C3b-cleaving factor I, factor H, a cofactor of factor I and a displacer of Bb from the AP C3bBb convertase, and C4b-binding protein, which interferes with the assembly of the CP/LP C4bC2a convertase.

A central role in the innate immune response and protection against staphylococcal infection is played by the central molecule of complement C3 but not factor B. In fact, mice with C3 deficiency show susceptibility to *S. aureus* septic arthritis and display impaired host clearance, presumably due to reduced opsonization and phagocytosis of bacteria (Na et al., 2016).

In turn, *S. aureus* secretes several peptides that interfere with the deposition of the complement on the bacterial surface. Indeed, the Staphylococcal binder of immunoglobulin Sbi helps to protect *S. aureus* from innate immune defense of the host and this effect is based on the ability to bind to the Fc region of IgG and the complement factor C3 in serum, promoting its futile consumption (Zhang et al., 1999; Burman et al., 2008). Furthermore, a secreted chemotaxis inhibitory protein of *S. aureus* (CHIPS) blocks function of the C5a and formylated peptide receptors needed for neutrophil chemotaxis (de Haas et al., 2004; Postma et al., 2004).

## Cells of host innate immunity

If microorganisms cross an epithelial barrier and start to replicate in the tissues of the host, they are promptly recognized, ingested and killed by the mononuclear phagocytes or macrophages that reside in the tissues. Another important family of phagocytes, neutrophils, are short-lived cells that are abundantly present in the blood but not in the tissues. Both macrophages and neutrophils play a key role in innate immunity because they can efficiently destroy many pathogens without the aid of adaptive immunity. In particular, neutrophils are a central player in the interaction between host and *S. aureus* (Newsom, 2008; Spaan et al., 2013). During infection, neutrophils leave the blood and migrate to the focus of infection in a multistep process mediated through adhesive interactions that are regulated by cytokines and chemokines (Spaan et al., 2013).

Cytokines are small proteins (~25 kDa) that are released by various cells in the body, in response to an activating stimulus and that induce responses through binding to specific receptors. They can act in an autocrine or in paracrine manner. Chemokines are a class of cytokines that have chemoattractant properties, inducing cells with the appropriate receptors to migrate toward the source of the chemokine. Chemokines mainly recruit leukocytes, in particular monocytes and neutrophils, and other effector cells from the blood to sites of infection. All the chemokines are related in amino acid sequence and their receptors are all integral membrane proteins containing seven membrane-spanning helices (Allen et al., 2007). Members of the chemokine family include CXC motif, in which two cysteine residues are separated by another amino acid. CXC chemokines bind to at least seven different CXC receptors (CXCR1-7) expressed on different cell types (Murdoch and Finn, 2000). The CXC chemokine receptor 2 (CXCR2), highly expressed on neutrophils, recognizes chemokines produced at the site of infection and plays an important role in antimicrobial host defenses, such as neutrophil activation and chemotaxis (Sekido et al., 1993; Chuntharapai et al., 1994; Eisele et al., 2011).

## *S. aureus* expresses A variety of proteases that play A role in pathogenesis

*Staphylococcus aureus* secretes a number of proteases, including two cysteine proteases (staphopain A, ScpA, and staphopain B, SspB), a serine protease (V8 or SspA), serine protease–like proteins (Spls) and a metalloproteinase (aureolysin, Aur). The protease genes are positively regulated by *agr* (accessory gene regulator) and negatively regulated by *sarA* (staphylococcal accessory regulator) (Shaw et al., 2004) and are organized into four distinct operons, encoding seven serine proteases (SspA and SplA-F), two cysteine proteases (ScpA and SspB), and Aur. The Aur, V8, SspB, and ScpA proteases are produced as zimogen, while the six Spl enzymes are active upon secretion. The Aur and ScpA precursors self-activate outside the cell, and SspA and SspB activation relies on a proteolytic cascade in which Aur processes V8 (Drapeau, 1978) and V8 cleaves and activates SspB (Massimi et al., 2002). Proteases of *S. aureus* were initially thought to play a role only in nutrient acquisition, however, evidence is emerging that they are crucially involved in the evasion of host immunity by interacting with neutrophils, Smagur et al. (2009) plasma proteins (Prokesová et al., 1992) and antimicrobial peptides (Sieprawska-Lupa et al., 2004; Table 1).

**Table 1:** **The main ***S. aureus*** secreted proteases**.

**Protease group**	**Function**	**References**
Staphopains A (ScpA)	Cleavage of neutrophils N-terminal domain of CXCR2	Laarman et al., 2012
Staphopains B (SspB)	Neutrophils CD31 cleavage	Smagur et al., 2009
V8 protease	Human IgG degradation	Prokesová et al., 1992
Aureolysin (Aur)	α1-protease inhibitor cleavage	Potempa et al., 1986
	Prothrombin activation	Wegrzynowicz et al., 1980
	Antimicrobial peptide LL-37 cleavage	Sieprawska-Lupa et al., 2004
	C3 cleavage	Laarman et al., 2011
Serine protease–like protein A (SplA)	Degradation of mucin 16	Paharik et al., 2016
Epidermin leader peptide processing serine protease (EpiP)	Cleavage of collagen and casein	Kuhn et al., 2014
Exfoliative toxins A (ETA) and B (ETB)	Degradation of desmoglein-1	Amagai et al., 2000, 2002

### Staphopains A (ScpA) and B (SspB)

Staphopains, two papain-like proteases of *S. aureus*, are both ~20 kDa proteins that have almost identical three-dimensional structures, despite sharing limited primary sequence identity. ScpA consists of two domains, refereed as L- and R- domain. The L-domain is built from the N-terminal part of the sequence and contains the active site helix that carries a nucleophilic cysteine. The R domain contributes the catalytic histidine and asparagine and is built around a size-stranded antiparallel pseudobarrel (Filipek et al., 2003).

Although there is limited data available on the virulence potential of staphopains *in vivo* models, experiments performed *in vitro* have demonstrated broad activity by these enzymes, including destruction of connective tissue, disturbance of clotting and kinin systems and direct interaction with host immune cells (Kantyka et al., 2011).

**Figure 3:**
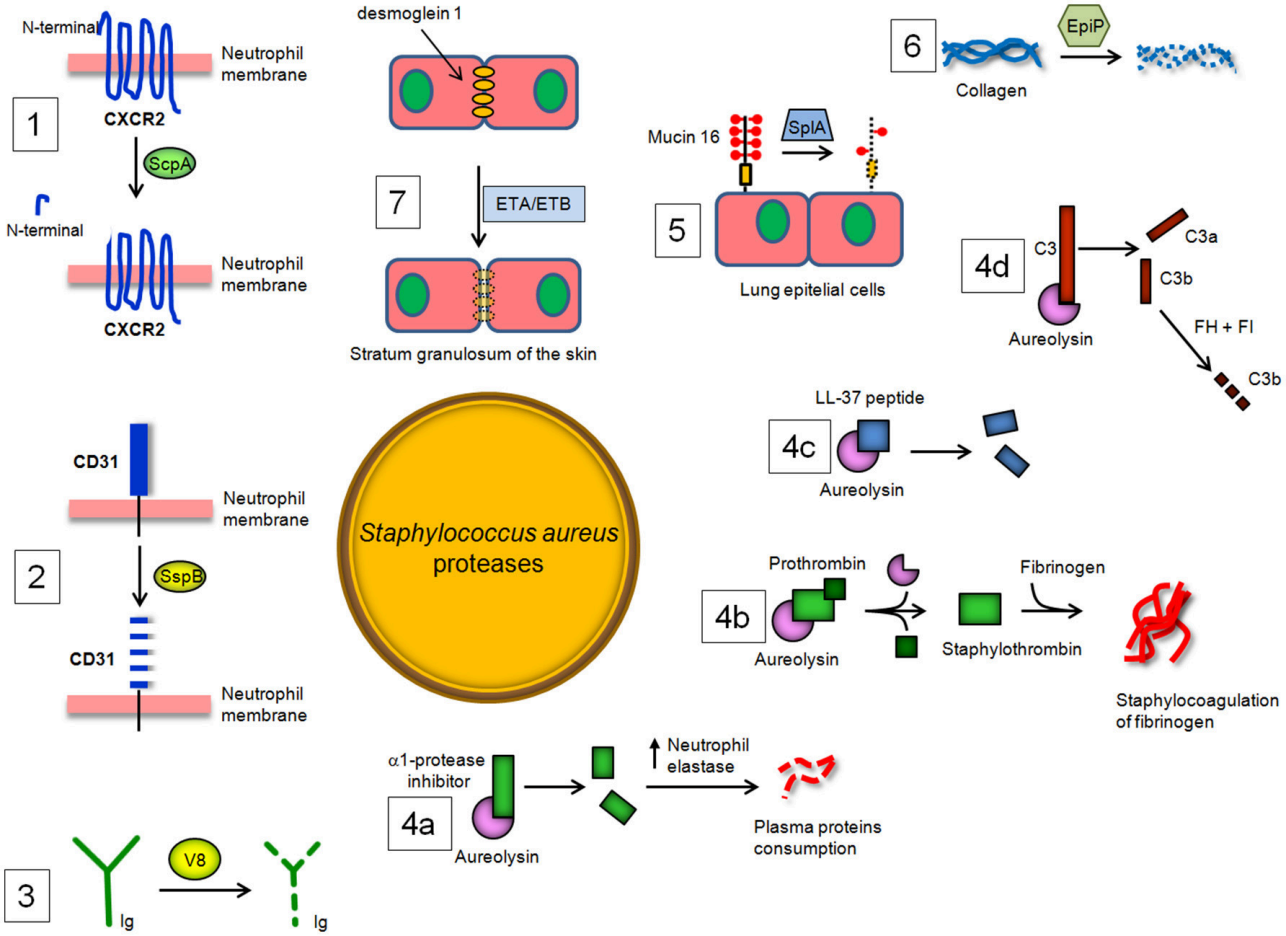
*****S. aureus*** proteases and their enzymatic targets in the host**. (1) Staphopain A (ScpA) cleaves the N-terminal domain of CXC receptor 2 (CXCR2) and impairs binding of CXC chemokines and consequent neutrophil activation and chemotaxis, while (2) Staphopain B (SspB) cleaves CD31, a member of the immunoglobulin superfamily, and dampens the functionality of neutrophils. (3) The cleaving of immunoglobulin classes by V8 protease leads to the avoidance of an immunological response and uncouples the ability of antibodies to link cell-surface antigen to immune effector cells. (4a) Aureolysin (Aur) cleaves and inactivates the α-1 protease inhibitor, resulting in deregulation of host elastase. Aur also (4b) activates prothrombin to thrombin and induces staphylocoagulation and (4c) neutralizes antibacterial peptide LL-37. Lastly, Aur (4d) can cleave C3, which compromises bacterial opsonization because the cleavage product C3b is promptly degraded by a complex of factor H and factor I. (5) SplA, a protease belonging to the Spls, is able to degrade mucin 6 on the surface of lung epithelial cells, promoting *S. aureus* colonization of subepithelial tissue. (6) Degradation by EpiP of collagens, essential components of the connective tissue, and (7) disruption by exfoliative toxin A (ETA) and B (ETB) of desmoglein-1, a desmosomal adhesion molecule that mediates intercellular adhesion in the stratum granulosum of the skin, also contributing to the spreading of *S. aureus* infection in the host tissues.

Neutrophils treated with ScpA do not respond to activation by CXCR2 chemokines after specific cleavage of the N-terminal domain and this effect can be neutralized by specific protease inhibitors. Moreover, ScpA inhibits neutrophil migration toward CXCR2 chemokines and tissue recruitment (Laarman et al., 2012; Figure 3). Despite the importance of these observations, it should be noted that, due to the complex and redundant meshwork of cytokine functions, it is difficult to extrapolate these *in vitro* findings to the situation in infected tissues.

Exposure of phagocytes (neutrophils and monocytes) to SspB impairs their antibacterial functions by repression of their chemotactic activity and determines the extensive clearance of SspB-treated cells by macrophages. SspB also cleaves on the surface of neutrophils CD31, a member of the immunoglobulin superfamily involved in the repulsive signaling pathway that discourages the predatory activity of macrophages. Consequently, the proteolytic activity of SspB dampens the functionality of neutrophils and explains the observed phagocytosis of SspB-treated neutrophils by monocyte-derived macrophages and the consequent staphylococcal colonization and spreading (Smagur et al., 2009; Figure 3).

### V8 protease

V8 protease is related to the pancreatic serine proteases (Prasad et al., 2004). The enzyme cleaves peptide bonds exclusively on the carboxyl side of glutamate (and aspartate, to a lesser extent) residues. Unlike the pancreatic serine proteases, V8 protease does not possess any disulphide bridges. This is a major evolutionary difference, as all pancreatic proteases have at least two disulphide bridges. V8 protease shows structural similarity with several other serine proteases, specifically the epidermolytic toxins A and B from *S. aureus* and trypsin, in which the conformation of the active site is almost identical (Prasad et al., 2004). V8 protease is also unique in that the positively charged N-terminus is involved in determining the substrate-specificity of the enzyme. V8 protease degrades all human immunoglobulin classes. Cleavage of IgG with V8 is associated with the partial loss of antigenic determinants and disturbance of the effector function due to the degradation of the Fc region, suggesting that V8 protease could uncouple the ability of antibodies to link cell-surface antigen to immune effector cells and may protect bacteria against defense mechanisms of the host (Prokesová et al., 1992 Figure 3).

### Aureolysin (Aur)

Aur is a zinc-dependent metalloprotease that belongs to the family of thermolysins. The structure of Aur has been determined, revealing a polypeptide chain of 301 amino acids which is folded into a β-pleated N-terminal domain and an α-helical C-terminal domain, a typical fold for the thermolysin family of metalloproteinases (Banbula et al., 1998).

*In vitro*, Aur has been shown to cleave and inactivate the α1-protease inhibitor, which is an endogenous protease inhibitor essential for controlling neutrophil serine protease elastase. Inactivation of α1-proteinase inhibitor results in the deregulation of the elastase and therefore may be important in the consumption of some plasma proteins by this enzyme during septicemia (Potempa et al., 1986). Notably, Aur activates prothrombin in human plasma and induces staphylocoagulation thereby suggesting a possible role of this protease in septic infections (Wegrzynowicz et al., 1980). Aur may also affect the stimulation of T and B lymphocytes by polyclonal activators and display inhibitory activity against immunoglobulin production by lymphocytes (Prokesová et al., 1991). Importantly, Aur contributes to staphylococcal immune evasion by cleavage of antimicrobial peptide LL-37 (Sieprawska-Lupa et al., 2004). Burlak et al have recently shown that Aur and other staphylococcal proteases can be expressed within the phagocytic vacuole following bacterial phagocytosis by human neutrophils (Burlak et al., 2007). This finding, along with information that isogenic *aur* mutant appears more efficiently killed by macrophages upon phagocytosis, indicates that Aur can protect staphylococci inside the phagocytes probably through resistance to antimicrobial peptide killing (Kubica et al., 2008). The action of Aur on complement component C3 has been also analyzed in detail, showing that Aur cleaves C3 to C3b, and the generated C3b is then rapidly degraded by the combination of factor H and factor I present in serum. As a result, bacteria are poorly opsonized with C3b, and this attenuates phagocytosis and killing by neutrophils (Laarman et al., 2011). In conclusion, Aur seems to facilitate not only the activation of V8 protease (Drapeau, 1978) but also to act in synergy with regulators of the complement system (Laarman et al., 2011; Figure 3).

### Serine protease–like proteins (Spls)

*Staphylococcus aureus* Spls are extracellular members of a group of 6 proteases (SplA-SplF) of unknown function expressed *in vivo* and encoded in one operon in the *S aureus* genome. To date, SplA, SplB, SplC, and SplD are the best-characterized Spl proteases in terms of biochemical and structural properties and show significant structural homology to V8 protease and epidermolytic toxins (Popowicz et al., 2006; Dubin et al., 2008; Stec-Niemczyk et al., 2009; Zdzalik et al., 2013). By way of example, SplA shows a chymotripsin-like fold and consists of two domains, each of which is made up of six antiparallel β strands folded into a β barrel. The active site of the enzyme is located at the interface of the two barrels and consists of the residues His, Asp and Ser conserved in all enzymatically active chymotrypsin-like proteases (Stec-Niemczyk et al., 2009). Spls elicit IgE antibody responses in most asthmatic patients. In healthy *S aureus* carriers and non-carriers, peripheral blood T cells elaborated TH2 cytokines after stimulation with Spls, as is typical for allergens. Thus, Spls can be considered as triggering allergens released by *S. aureus* opening prospects for diagnosis and causal therapy of asthma (Stentzel et al., 2016). Moreover, Spls are required for *S. aureus* to cause disseminated lung damage in a rabbit model of pneumonia. In particular, SplA is able to cleave mucin 16, a glycosylated cell surface protein from the human lung cell line CalU-3, suggesting that removal of this protein might promote *S. aureus* invasion and spreading of host tissues. Finally, analysis of the secreted and surface proteins expressed by *S. aureus* USA 300 and *slp* mutant strains revealed many bacterial proteins altered in abundance, suggesting a role of these proteases on the modulation of virulence factor production (Paharik et al., 2016). It remains to be determined whether Spls, with their proteolytic potential, have an impact on the activities of the immune defense mechanisms of the host (Figure 3).

### EpiP

A homolog of an *S. epidermidis* protein annotated as an epidermin leader peptide processing serine protease (EpiP) (Geissler et al., 1996) has been identified and characterized in *S. aureus* (Kuhn et al., 2014). The *S. aureus* EpiP is released into the extracellular milieu and expressed as zymogen that can be cleaved through an autocatalytical intramolecular mechanism. The protein acts as a serine protease and is capable of cleaving both collagen and casein (Kuhn et al., 2014; Figure 3). The *epiP* gene contains a peptidase-S8 domain that is present in subtilisin-like serine proteases and in the *Streptococcus pyogenes* homolog SpyCEP protease. It is well established that SpyCEP inactivates IL-8 catalyzing its C-terminal cleavage (Edwards et al., 2005). As consequence, SpyCEP impairs the recruitment of neutrophils at the site of infection and bacterial clearance (Zinkernagel et al., 2008). Given the essential role of neutrophils in fighting bacterial infection (Andrews and Sullivan, 2003; Döhrmann et al., 2016) one can assume that EpiP could display a pathogenic activity similar to SpyCEP.

### Exfoliative toxins A (ETA) and B (ETB)

Skin is a critical protective barrier against several external agents, such as bacteria, allergens, ultraviolet radiation and mechanical insult. *S. aureus* possesses biochemical tools to penetrate and injure skin. A direct breakage of the skin involves the secreted staphylococcal exfoliative toxins A and B (ETA/ETB) which cause blister formation in staphylococcal scalded skin syndrome (SSSS) and bullous impetigo. ETA and ETB are serine proteases with a similar overall structure including the positions of key residues within the active site (Vath et al., 1999). ETA/ETB specifically cleave by identical mechanisms desmoglein 1, a desmosomal adhesion molecule that mediates intercellular adhesion in the stratum granulosum of the skin, without affecting desmoglein 3 or E-cadherin (Amagai et al., 2000, 2002; Figure 3). In SSSS, *S. aureus* is present in distant foci, such as the nose, pharynx or conjunctiva, and toxin produced by *S. aureus* can spread through the bloodstream and cause exfoliation in remote sites, whereas in bullous impetigo, a localized form of SSSS, it is present only in the lesions. ETs share both cleavage site on desmoglein 1 and high degree sequence similarity with V8 protease (Dubin, 2002). Therefore, it has been speculated that ETs and V8 might act together to disrupt desmoglein 1 and compromise the stability and barrier function of the skin (Katayama et al., 2013).

In a variant of the above strategy, *S. aureus* cells capture activated host proteases that directly cleave essential components of the host defense mechanisms. For example, the cell wall-anchored protein clumping factor A binds to complement regulator factor I and increases factor I-driven cleavage of complement component C3b (Hair et al., 2008).

Likewise, surface protein SdrE enhances recruitment of the complement regulator factor H (FH). SdrE-bound FH retains cofactor activity for factor I-mediated cleavage of C3b and this results in down-regulation of complement effectors and in increased protection from neutrophil killing (Sharp et al., 2012).

## *S. aureus* expresses secreted proteins that modulate host protease activity

*Staphylococcus aureus* is specialized in handling host proteins that are involved in the complement system, coagulation cascade and fibrinolysis cascade (Chavakis et al., 2005; Imamura et al., 2005; Nizet, 2007). Proteins that play a role in these pathways circulate in biological fluids or are in the extracellular matrix as inactive zymogen that can be activated upon interaction with specific secreted staphylococcal proteins. Alternatively, *S. aureus* secretes specific proteinaceous inhibitors of host serine proteases that play key roles in immune defense. In both cases, these proteins positively affect bacterium pathogenicity *in vivo* (Stapels et al., 2014; Table 2).

**Table 2:** **The main ***S. aureus*** modulators of host proteases**.

**Modulator**	**Function**	**References**
**ACTIVATOR**		
Coagulase(Coa) and von Willebrand factor-binding protein (vWbp)	Prothrombin activation	Friedrich et al., 2003; Bjerketorp et al., 2004; Kroh et al., 2009
Staphylokinase (SAK)	Plasminogen activation	Bokarewa et al., 2006
α-toxin	ADAM10 activation	Wilke and Bubeck Wardenburg, 2010; Inoshima et al., 2011
**INHIBITOR**		
Extracellular adherence protein (Eap)	CP/LP C3 proconvertase inhibition	Woehl et al., 2014
Superantigen-like proteins 1 (SSL1) and 5 (SSL5)	Inhibition of metalloproteases	Bestebroer et al., 2007; Chung et al., 2007
*S. aureus* collagen adhesin (Cna)	Inhibition of C1 complex formation	Kang et al., 2013
Staphylococcal Complement Inhibitor (SCIN)	Inhibition of AP C3 convertase	Rooijakkers et al., 2005a
Extracellular fibrinogen binding protein (Efb)	Inhibition of AP C3 convertase	Chen et al., 2010
Serine aspartate glycosyltransferases A (SdgA) and B (SdgB)	Inhibition of human neutrophil-derived catepsin G	Hazenbos et al., 2013

### Staphylococcal activators of host proteases

#### Coagulase(Coa) and von Willebrand factor binding protein (vWbp)

**Figure 4:**
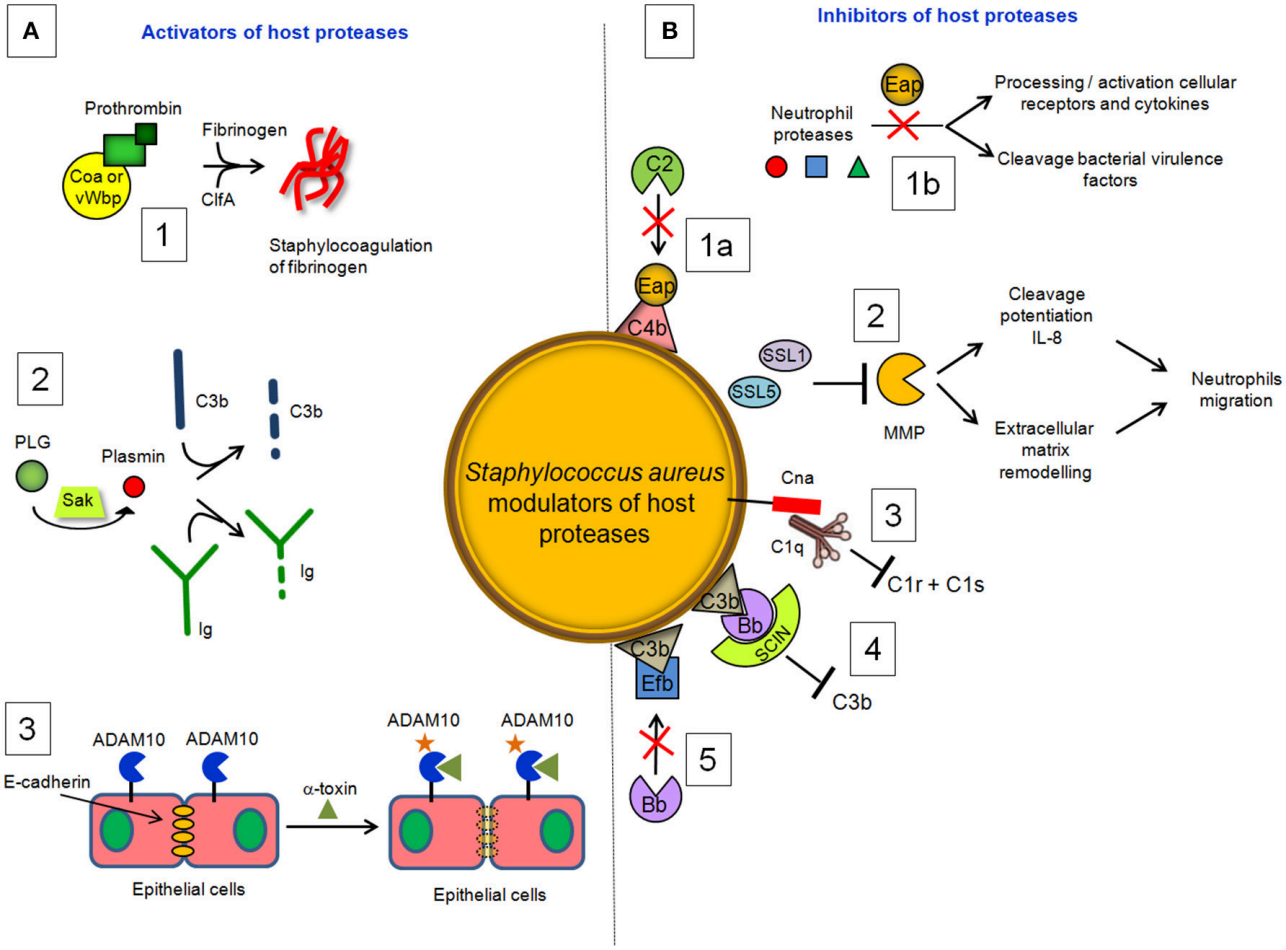
*****S. aureus*** modulators of host proteases. (A)** Activators. (1) Coa and vWbp activate prothrombin to cleave fibrinogen, whereas clumping factor A allows staphylococci to associate to form fibrin cables. (2) The plasminogen (PLG) binding protein staphylokinase (SAK) activates the zymogen to the active protease plasmin, which can degrade complement opsonin C3b and the immunoglobulin Fc domain. (3) α-toxin binds to the ADAM10 receptor to disrupt the physiological barrier functions of tissues such skin. **(B)** Inhibitors. (1a) Extracellular adherence protein (*Eap*) disrupts formation of the CP/LP C3 proconvertase (C4bC2) by preventing C4b from binding to C2, inhibiting formation and deposition of C3b on the surface of *S. aureus* cells. (1b) Eap specifically inhibits neutrophil serine proteases elastase, proteinase 3 and cathepsin G, by blocking processing and activation of cellular receptors and chemokines and cleavage of bacterial virulence factors. (2) Superantigen-like protein 1 (SSL1) and 5 (SSL5) prevent matrix metalloprotease (MMP)-induced cleavage of IL-8, a chemokine produced by macrophages and other cell types that induces neutrophil chemotaxis, and inhibit the remodeling of extracellular matrix and the consequent migration of neutrophils through collagen. (3) Collagen adhesin (Cna) blocks the association of C1q bound to immunoglobulin with complement component C1r and inhibits the classic pathway formation. (4) The secreted peptide Staphylococcal Complement Inhibitor (SCIN) stabilizes C3bBb convertase of the alternative pathway in an inactive form, preventing the production of C3a, C3b, and C5a. (5) The extracellular fibrinogen binding protein (Efb) induces a conformational change in C3b in a way that impairs its interaction with complement factor B and formation of the active C3 convertase of the alternative pathway.

Coagulase (Coa) is an *S. aureus* protein comprised of the D1D2 domain in the N-terminal part involved in prothrombin binding, a linker domain and a repeat domain composed of tandem repeats of a 27-residue-long-segment in the C-terminal part that binds to fibrinogen. Coa promotes blood coagulation by activating prothrombin through insertion of the Ile1-Val2 N terminus of the Coa D1D2 domain into the Ile16 pocket of prothrombin, inducing a functional active site in the zymogen through conformational change (Friedrich et al., 2003). The Coa/prothrombin complex then specifically recognizes fibrinogen and converts it into fibrin (Panizzi et al., 2006). Von Willebrand factor-binding protein) (vWbp) is another secreted *S. aureus* coagulase which, in addition to binding vWF, associates with prothrombin to convert fibrinogen to fibrin (Friedrich et al., 2003; Bjerketorp et al., 2004; Kroh et al., 2009). vWbp displays sequence homology to the Coa D1D2 domain, whereas its C-terminal region lacks the linker segment and repeat domain of Coa, which are replaced by unique vWF and fibrinogen binding sites (Bjerketorp et al., 2002; Cheng et al., 2010). When suspended in human or animal plasma, staphylococci can form large aggregates. Coa, vWbp and clumping factor A are required for bacterial agglutination: Coa and vWbp activate prothrombin to cleave fibrinogen, whereas clumping factor A allows staphylococci to associate to form fibrin cables (McAdow et al., 2011; Walker et al., 2013; Figure 4). The formation of fibrin networks protects the bacterium from neutrophil and phagocytic clearance, and facilitates the pathogenesis of lethal blood stream infection in mice (Walker et al., 2013).

Activation of prothrombin by coagulases also induces direct cleavage of complement component C3, as well as its activation fragments. Moreover, thrombin can cleave C5 into C5a, which occurs independently of C3 and therefore represents a bypass of the three traditional complement-activation pathways (Rittirsch et al., 2008).

#### Staphylokinase (SAK)

Staphylokinase (SAK), is a 136 aa long bacteriophage-encoded protein expressed by lysogenic strains of *S. aureus* (Peetermans et al., 2016). SAK is both present in the cell culture environment and associated with the cell surface of staphylococci. Present understanding of the role of SAK during bacterial infection is based on its interaction with the host proteins. Binding of SAK to human antibacterial peptides α defensins and LL-37 abolishes their bactericidal properties, which makes SAK a useful tool for staphylococcal resistance to host innate immunity (Jin et al., 2004; Braff et al., 2007). The main SAK activity is related to its ability to bind and convert plasminogen (PLG) to active, broad spectrum proteolytic enzyme plasmin (Bokarewa et al., 2006). Unlike direct human PLG activators, such as tissue plasminogen activator (t-PA) (Lijnen and Collen, 1988) and urokinase (UK) (Vassalli et al., 1985; Blasi et al., 1986), SAK does not have any proteolytic activity of its own but acts by forming a 1:1 stoichiometric complex with plasmin and changing its substrate specificity to activate PLG (Peetermans et al., 2016). The activation of PLG by SAK is facilitated by the ability of staphylococci to capture PLG at the bacterial surface through surface-expressed proteins, such as FnBPA and FnBPB (Pietrocola et al., 2016). By activating human PLG into plasmin at bacterial surface, SAK creates bacterium-bound serine protease activity that induces fibrin specific thrombolysis in human plasma (Collen and Lijnen, 2005) and leads to the degradation of two major opsonins, human immunoglobulin G (IgG) and human C3b (Rooijakkers et al., 2005b; Figure 4). The finding that SAK-induced PLG activation prevents *S. aureus* biofilm formation and/or detachment of existing biofilm through cleavage of the major biofilm component fibrin strongly suggests a crucial role of this protein in controlling biofilm formation (Kwiecinski et al., 2016). Staphylococcal bound plasmin has been also shown to cleave the 55kDa pro-matrix metalloprotease 1 into the mature 42 kDa active matrix metalloprotease 1, one of the major interstitial collagenase (Santala et al., 1999). This effect possibly provides a direct cue for leukocyte migration and activation.

The findings that clinical isolates of skin and mucosal origin expressing high levels of SAK show a more efficient invasion of internal organs than strains expressing a low level of SAK, and the finding that in animal sepsis wild type strains show an increased bacterial load compared to the *sak* isogenic mutants (Bokarewa et al., 2006) supports evidence that SAK is an important staphylococcal virulence factor. Although SAK is present in the vast majority of *S. aureus* strains causing human infections, the frequency of staphylococcal expression varies between 4 and 100% in different collections of *S. aureus* isolates (Declerck et al., 1994; Jin et al., 2003). Furthermore, a study on the course of haemogenous staphylococcal sepsis induced by the SAK-producing strain revealed no difference in mortality or weight loss compared to the isogenic strain incapable of producing SAK (Kwieciński et al., 2010). These observations make the role of SAK as a “critical” virulence factor in *S. aureus* diseases questionable.

#### α-toxin

Exposure to *S. aureus* pore-forming α-toxin, also known as α-hemolysin, can cause cellular death by necrosis, apoptosis, or pyroptosis, through activation of different cellular pathways (Essmann et al., 2003; Craven et al., 2009). α-toxin also binds to the receptor ADAM10 in alveolar epithelial cells. Binding of α-toxin to ADAM10 results in upregulation of ADAM10 metalloprotease activity, with the consequent cleavage of E-cadherin, a protein engaged in homotypic intercellular interactions in adherens junctions. Cleavage is associated with disruption of epithelial barrier function, increased staphylococcal invasion and a lethal acute lung injury of mice (Inoshima et al., 2011); (Wilke and Bubeck Wardenburg, 2010; Figure 4). These studies demonstrate that α-toxin disrupts barriers not only by lysing cells but also by the more subtle mechanism of activating a host protease.

### Staphylococcal factors that interfere with host protease activities

Among the anti-bacterial functions, neutrophils produce serine proteases including proteinase 3, cathepsin G and elastase. Neutrophils also secrete matrix metalloproteases that regulate the degradation of extracellular matrix components (Nagase et al., 2006) and turnover of non-matrix substrates including cytokines, chemokines, growth factors and receptors (Parks et al., 2004; Rodríguez et al., 2010). Furthermore, it is well known that the complement system is a proteolytic cascade where serine proteases activate each other through limited proteolysis in a strictly ordered manner. Therefore, bacterial mechanisms that interfere with such protease activities may be potentially important to safeguard efficient host tissue colonization.

#### Extracellular adherence protein (Eap)

*Staphylococcus aureus* secretes a multifunctional protein named extracellular adherence protein (Eap). Mature Eap molecule is ~50–70 kDa and comprises four to six tandem repeats of ~97 residue domain joined by short, 9–12 residue linker region (Jönsson et al., 1995; Geisbrecht et al., 2005). Eap shows a unique ability to form protein–protein interactions with an array of ligands, including a bacterial cell surface-retained phosphatase (Flock and Flock, 2001), host extracellular matrix molecules, such as collagen, fibronectin, and laminin (Bodén and Flock, 1992; McGavin et al., 1993; Palma et al., 1999), and the pro-inflammatory mammalian surface adhesin ICAM-1 (Chavakis et al., 2002). Notably, Eap disrupts formation of the CP/LP C3 proconvertase (C4bC2) by preventing C2 from binding to C4b (Figure 4). Hence, Eap inhibits deposition of C3b on the surface of *S. aureus* cells and significantly diminishes the extent of *S. aureus* opsonophagocytosis and killing by neutrophils (Woehl et al., 2014). Eap also inhibits the activity of elastase, proteinase 3 and cathepsin G, a class of neutrophil serine proteases (NSPs) stored within the azurophilic granules (Pham, 2006). Crystallographic studies by Stapels et al. demonstrated that Eap behaves as a protease inhibitor which occludes the catalytic cleft of neutrophil serine proteases and inhibits their activity (Stapels et al., 2014).

Notably, upon neutrophil activation, neutrophil serine proteases either enter the nucleus to regulate extracellular trap formation (Papayannopoulos et al., 2010) or are released into the extracellular milieu to cleave bacterial virulence factors (Weinrauch et al., 2002) and/or chemokines and receptors (Korkmaz et al., 2010). Therefore, Eap can effectively counteract crucial antibacterial activity associated to neutrophil proteases.

#### Superantigen-like proteins 1 (SSL1) and 5 (SSL5)

Matrix metalloproteases constitute a large family of structurally related, zinc-dependent proteases. They facilitate immune cell migration as a consequence of breakdown of extracellular matrix components and potentiate the activity of chemokines, enhancing inflammation and aiding bacterial clearance. Matrix metalloprotease 8 (neutrophil collagenase) and 9 (neutrophil gelatinase B) are highly expressed and produced by neutrophils, stored in secondary and tertiary granules and secreted upon cell activation (Parks et al., 2004).

To counteract the activity of these proteases, *S. aureus* secretes superantigen-like proteins 1 (SSL1) and 5 (SSL5), which are members of the SSL family. The number of SSL members expressed in the staphylococcal cells varies from 7 to 11, depending on the strain of *S. aureus* (Fitzgerald et al., 2003). SSL proteins are characterized by the presence of an N-terminal-barrel globular domain linked to the C-terminal-grasp domain, which is a structural feature common to TSST-1 (toxic shock syndrome tovin-1) and enterotoxins (Williams et al., 2000).

SSL1 and SSL5 prevent matrix metalloprotease-induced cleavage and potentiation of IL-8, a chemokine produced by macrophages and other cell types that induces neutrophil chemotaxis, and inhibit the remodeling of extracellular matrix and migration of neutrophils through collagen (Bestebroer et al., 2007; Chung et al., 2007; Figure 4). Therefore, through matrix metalloprotease-inhibition, SSL1 and SSL5 limit neutrophil activation, chemotaxis, and migration, all critical neutrophil functions in bacterial clearance (Koymans et al., 2016).

#### Staphylococcus aureus collagen adhesion (Cna) and staphylococcal complement inhibitor (SCIN)

The early event of the CP activation involves binding of C1q to two molecules each of the proenzymes C1r and C1s, forming the C1 complex C1q:C1r_2_C1s_2_. Once activated, the serine protease C1s cleaves C4 and then C2 to generate two large fragments which combine together to form the C3 convertase of the CP. The *S. aureus* collagen adhesin (Cna) interacts also with C1q resulting in the inhibition of its interaction with C1r. Consequently, C1r_2_C1s_2_ is displaced from C1q and activation of the CP is not allowed (Kang et al., 2013; Figure 4). Along this line the secreted peptide Staphylococcal Complement Inhibitor) (SCIN) associates with and stabilizes C3bBb convertase of the alternative pathway in an inactive form, thereby preventing the production of C3a, C3b, and C5a (Rooijakkers et al., 2005a; Figure 4). Cna and SCIN may be important components of a more general evasion strategy of this remarkable pathogen.

#### Extracellular fibrinogen binding protein (Efb)

The extracellular fibrinogen binding protein Efb is another innate immune evasion molecule secreted by *S. aureus*, which is reported to block platelet aggregation (Shannon and Flock, 2004; Shannon et al., 2005), delay wound healing in a rat wound infection model (Palma et al., 1996) and inhibit neutrophil adherence to immobilized fibrinogen (Ko et al., 2011). Efb protein has a disordered N-terminal fibrinogen-binding region and a folded C3-binding domain in the C-terminal region (Hammel et al., 2007). Efb acts as an allosteric inhibitor by inducing conformational changes in factor C3b that propagate across several domains and influence functional regions far removed from the Efb binding site. Consequently it impairs the interaction of C3b with complement factor B and the formation of active C3 convertase (Chen et al., 2010; Figure 4).

#### Serine aspartate glycosyltransferases A (SdgA) and B (SdgB)

Crucial for staphylococcal adherence and colonization of host tissues is a family of staphylococcal cell wall-anchored proteins containing several repeats of serine-aspartate (SD) residues located between the N-terminal ligand-binding A domain and a C-terminal LPXTG motif (Foster et al., 2014). The prototype members of this family are clumping factor A and B, which are important virulence factors mediating the attachment of *S.aureus* to several extracellular matrix components (Foster et al., 2014). Two recently identified *S. aureus* glycosylases, SdgA and SdgB, are responsible for direct concerted glycosylation of the SD moieties of these proteins. Although the precise role of SD repeat glycosylation is still to be defined, it has been suggested that this event could render bacterial proteins invulnerable to proteolysis by human neutrophil-derived catepsin G, prevent their degradation and preserve the structural and functional integrity of these important virulence factors (Hazenbos et al., 2013).

## Discussion and perspectives

The fact that proteases and regulators of host proteases are secreted abundantly by almost all the strains of *S. aureus*, and the observation that mutants lacking proteases show a decrease in abscess formation and impairment during organ invasion, indicate that they play a crucial role as virulence factors (Kolar et al., 2013). Important progress has been made over recent decades in the identification and understanding of the functional role of *S. aureus* proteases and secreted factors that regulate host proteolytic activities. Specific advancements include the assessment of the multiple roles of these factors in the modulation of the complement system and prevention of phagocytosis. It has been proven that *S. aureus* proteases cleave tissue adhesion molecules allowing transition from adhesive to invasive phenotype and the consequent dissemination and spreading of bacterial infection. Furthermore, incubation of human serum with a combination of Aur, protease V8 and cysteine proteases staphopain A and B causes complete inhibition of all complement pathways. This results in a drastic decrease in the haemolytic activity of the serum and suggests that the concerted action of the four proteases is important for pathogen-mediated evasion of the human complement system (Jusko et al., 2014).

Despite the acquisition of these important insights, many structural and functional aspects of these factors remain unknown. For example, the expression and structural analysis by X-ray crystallography of many of these factors, alone or in combination with specific targets, are still lacking. Similarly, we have just begun to explore the role of these proteins in high quality animal studies and how each factor relates to the complex sequence of events in the initiation and progression of infection. Finally, new advances in the antigenic properties of these factors represent an important basis for the development of promising approaches for managing staphylococcal disease. The acquisition of this wealth of information in the short term would provide opportunities to develop specific synthetic drugs targeting one or more proteases or inhibiting the activity of staphylococcal modulators of host proteases. On this matter, it has been suggested that Eap “might serve as a template for developing a new class of synthetic inhibitors of neutrophil serine proteases” to treat inflammatory disorders like cystic fibrosis and emphysema, where neutrophil serine proteases play a significant role (Stapels et al., 2014). Although in a different contest, this example illustrates how the understanding of staphylococcal virulence factors might contribute to the development of therapeutics in medicine.

Moreover, the consistency between the positive regulation of *S. aureus* proteases by the accessory gene regulator *agr* (Shaw et al., 2004; Novick and Geisinger, 2008) and the observation that selective chemical inhibition of *agr* quorum sensing promotes and strengthens the host defense immunological system (Sully et al., 2014; Tsuchikama et al., 2017) paves the way for the development of new therapeutic strategies against *S. aureus* diseases.

Prospectively, antibodies against such *S. aureus* factors targeting elements of the immune system could restore host defense mechanisms and have clinical utility as adjunctive agents enhancing antibiotic efficacy in severe invasive diseases. Approaches to *S. aureus* vaccine development have so far been unsuccessful (Pozzi et al., 2015). This failure can be partly explained by the broad spectrum of the bacterium's immunevasive attributes that neutralize phagocytic killing of bacteria. Therefore, development of an effective *S. aureus* vaccine should consider the important role of virulence factors produced by this pathogen.

## Author contributions

PS and GP wrote the manuscript, GP, GN, and SR prepared the references, and PS finalized the manuscript.

## Funding

This work was supported by Fondazione CARIPLO (Grant Vaccines 2009-3546) to PS.

### Conflict of interest statement

The authors declare that the research was conducted in the absence of any commercial or financial relationships that could be construed as a potential conflict of interest.
